# Supplementary material for: A high-resolution mRNA expression time course of embryonic development in zebrafish
Source: eLife. 2017 Nov 16;6:e30860. doi: 10.7554/eLife.30860 (PMC5690287; doi:10.7554/eLife.30860)
Supplement: Supplementary file 6. [file elife-30860-supp6.zip › biolayout-clusters-files/Cluster114-genes.html]

Cluster114


# Cluster114: Genes

| Ensembl ID | Gene Name | Chr | Start | End | Biotype |
| --- | --- | --- | --- | --- | --- |
| ENSDARG00000074572 | G3BP2 (1 of many) | 23 | 44224948 | 44246512 | protein\_coding |
| ENSDARG00000019765 | LUC7L2 | 1 | 54491479 | 54505437 | protein\_coding |
| ENSDARG00000013382 | cwc15 | 5 | 23072528 | 23079646 | protein\_coding |
| ENSDARG00000030789 | ddx18 | 9 | 38504671 | 38542425 | protein\_coding |
| ENSDARG00000070108 | dek | 16 | 41065131 | 41080602 | protein\_coding |
| ENSDARG00000042252 | eif4h | 15 | 4594119 | 4625781 | protein\_coding |
| ENSDARG00000098627 | gspt1l | 3 | 36504196 | 36522452 | protein\_coding |
| ENSDARG00000069422 | nhp2 | 21 | 30365089 | 30372509 | protein\_coding |
| ENSDARG00000032175 | puf60a | 2 | 32670752 | 32688636 | protein\_coding |
| ENSDARG00000023299 | snu13b | 12 | 10405942 | 10409335 | protein\_coding |
| ENSDARG00000035559 | tp53 | 5 | 23582427 | 23594005 | protein\_coding |
